# NTF3 Is a Novel Target Gene of the Transcription Factor POU3F2 and Is Required for Neuronal Differentiation

**DOI:** 10.1007/s12035-018-0995-y

**Published:** 2018-03-16

**Authors:** Yi-Mei J. Lin, I-Lun Hsin, H. Sunny Sun, Shankung Lin, Yen-Ling Lai, Hsuan-Ying Chen, Ting-Yu Chen, Ya-Ping Chen, Yi-Ting Shen, Hung-Ming Wu

**Affiliations:** 1 Institute of Biomedical sciences, National Chung Hsing University, Taichung City, Taiwan; 2 Inflammation Research & Drug Development Center, Changhua Christian Hospital, No. 135 Nanxiao Street, Changhua, 500 Taiwan; 3 Institute of Molecular Medicine, National Cheng Kung University, Tainan, Taiwan; 4 Graduate Institute of Acupuncture Science, China Medical University, Taichung, Taiwan; 5 Department of Neurology, Changhua Christian Hospital, Changhua, Taiwan

**Keywords:** POU3F2, Neuron differentiation, Neurotrophin 3

## Abstract

POU-homeodomain transcription factor POU3F2 is a critical transcription factor that participates in neuronal differentiation. However, little is known about its downstream mediators. Here genome-wide analyses of a human neuronal differentiation cell model, NT2D1, suggested neurotrophin-3 (NTF3), a key mediator of neuronal development during the early neurogenic period, as a putative regulatory target of POU3F2. Western blot, cDNA microarray, and real-time quantitative PCR analyses showed that POU3F2 and NTF3 were upregulated during neuronal differentiation. Next-generation-sequence-based POU3F2 chromatin immunoprecipitation-sequencing and genome-wide in silico prediction demonstrated that POU3F2 binds to the *NTF3* promoter during neuronal differentiation. Furthermore, unidirectional deletion or mutation of the binding site of POU3F2 in the *NTF3* promoter decreased promoter-driven luciferase activity, indicating that POU3F2 is a positive regulator of *NTF3* promoter activity. While NTF3 knockdown resulted in decreased viability and differentiation of NT2D1 cells, and POU3F2 knockdown downregulated NTF3 expression, recombinant NTF3 significantly rescued viable neuronal cells from NTF3- or POU3F2-knockdown cell cultures. Moreover, immunostaining showed colocalization of POU3F2 and NTF3 in developing mouse neurons. Thus, our data suggest that *NTF3* is a novel target gene of POU3F2 and that the POU3F2/NTF3 pathway plays a role in the process of neuronal differentiation.

**Electronic supplementary material:**

The online version of this article (10.1007/s12035-018-0995-y) contains supplementary material, which is available to authorized users.

## Introduction

POU-homeodomain transcription factor POU3F2 (also known as BRN2) is one of four closely related Class III POU proteins that are predominantly expressed in the central nervous system (CNS) and are involved in patterning the embryonic brain [1, 2]. POU3F2 was originally found in neuronal cells but has also been detected in oligodendrocytes and melanocytes [2–4]. This brain-specific transcription factor is upregulated in the progenitor cells in the subventricular zone, intermediate zone, and outer layer of the neocortex during the early stages of embryonic brain development [5, 6], when POU3F2 contributes to neural formation and cell fate determination [7] and also regulates cortical neural migration [8], neurogenesis, and positioning of cortical neurons [9, 10]. Targeted inactivation or deletion of the *Pou3f2* gene in mice results in complete loss of the development of specific neuronal lineages in paraventricular nuclei and supraoptic nuclei in the hypothalamus [2]. Forced expression of POU3F2 and other neuronal transcription factors (ASCL1, MYT1L, and NEUROD1) has been shown to induce neuronal fate in pluripotent stem cells and to convert fetal and postnatal human fibroblasts or somatic cells into functional neurons [11–15]. More recently, Urban and colleagues demonstrated that POU3F2 was essential for retinoic acid-induced neuronal differentiation from mouse embryonic stem cells by regulating a set of target genes via binding to the genomic locus Zic 1 [16]. These results indicate that POU3F2 is a critical transcription factor in the conversion of embryonic stem cells into neurons and glial cells; however, little is known about the neurogenic genes it regulates.

Neurotrophin-3 (NTF3), a member of the neurotrophin family that includes nerve growth factor, brain-derived neurotrophic factors, and neurotrophin 4/5, has emerged as a key mediator of neuronal development during the early neurogenic period as well as throughout adulthood. NTF3 has been shown to maintain synapse function and synaptogenesis [17, 18]. Neurotrophins bind selectively to specific tyrosine receptor kinases to relay signals in various signaling pathways [19]. The binding of NTF3 to TrkC induces PI3K/Akt and RAS/ERK signaling pathways that regulate cell growth, survival, and differentiation [19, 20]. Functional deficiency in NTF3 has been shown to cause severe neuronal deficits and early postnatal death in mice [21]. Furthermore, motor neurons derived from conditional *Ntf3-*knockout embryos showed increased apoptosis and abnormal projection of the central-branch-innervating motor neuron, suggesting that NTF3 is critically important for survival and axonal projection of neurons [22].

In this study, we applied a genome-wide integrated analysis of the human NT2D1 cell model to identify putative POU3F2 target genes. When properly induced, NT2D1 cells are capable of differentiation into neuronal cells [23]. Our strategy, which combined computational-based and next-generation-sequencing-based genome-wide POU3F2-binding-site screening (ChIP-seq) to investigate the neuronal differentiation process, successfully identified several hundred putative genes that are targeted by POU3F2 during neuronal differentiation. One of the putative genes was *NTF3*. Accordingly, we have investigated whether POU3F2 regulated NTF3 expression and the role of the POU3F2/NTF3 pathway in the early stages of neuronal development using a human pluripotent embryonic carcinoma cell line NTERA2 cl.D1 (NT2D1). Our data show that *NTF3* is a novel target gene of POU3F2 and provide new insight into the mechanisms through which POU3F2/NTF3 regulates neuronal differentiation.

## Materials and Methods

### Animals

Timed-pregnant adult C57BL/6J female mice were purchased from the National Lab Animal Center (Taiwan). All procedures were approved by the Animal Care and Use Committee of Changhua Christian Hospital.

### Primary Neuron-Enriched Cultures

Mouse primary neuron-enriched cultures were prepared using a previously described protocol [24]. Briefly, cerebral tissues were dissected from mouse embryonic day (E) 14.5. Cells were dissociated by gentle mechanical trituration and immediately seeded at a density of 5 × 10^5^ cells/well in 24-well plates pre-coated with poly D-lysine (20 μg/ml). Plates were maintained at 37 °C in a humidified atmosphere of 5% CO_2_ and 95% air. Forty-eight hours after seeding, cytosine β-D-arabinofuranoside (10 μM) was added to prevent glial proliferation and was then removed 24 h later. The cultures were subsequently maintained in serum-free neurobasal medium (Invitrogen, Carlsbad, CA, USA) supplemented with 2% B27 and 2 mM glutamine and were given fresh medium every 3–4 days. Neuron-enriched cultures were 98% pure, and microglia-depleted cultures were 95% pure.

### NTERA2 cl.D1 Cell Line

Human pluripotent embryonic carcinoma NTERA2 cl.D1 (NT2D1) cells (ATCC, CRL1973) were cultured in Dulbecco’s Modified Eagle’s Medium (DMEM) supplemented with 10% fetal bovine serum (FBS). The cells were incubated at 37 °C in a humidified atmosphere containing 5% CO_2_ and used over a restricted culture period of 10 passages.

### Neuronal Differentiation of NT2D1 Cells

Differentiation of NT2D1 cells was conducted as described previously [23, 25, 26]. Briefly, confluent NT2D1 were first cultured in pre-induction DMEM medium supplemented with 20% FBS and 10 ng/ml bFGF (Gibco, 13256-029) for 24 h. Cells were then incubated in neuron induction medium (GlutaMax DMEM, 10 ng/ml bFGF, 10 ng/ml PDGF-BB, 100 μM BHA, 10 μM forskolin, 2 mM valproic acid, 25 mM KCl, 2% DMSO, and 1× B27 supplement) for another 24 h to induce neuronal differentiation. For the NTF3 treatment experiment, NTF3 recombinant protein (ProSpec, CYT-257) was added to the neuron induction medium during the neuron differentiation step.

### Genome-wide In Silico Prediction of Putative POU3F2 Binding Sites and Bioinformatic Promoter Analysis of NTF3 Promoter Sequences

We applied computer-based search tools to search for potential target genes of POU3F2. One was “The Binding Element Searching Tools (The BEST; NCKU Bioinformatics Center),” which was used to build a customized hidden Markov model (HMM) based on a set of sequences containing known POU3F2 binding sites. This model was then used to screen the promoter regions of all known human genes. The other one was the “AnGEL genome-wide CRM Searches” in the Transcription Element Search System (TESS) web server, which was used to scan the promoter sequences of all identified human genes for the presence of the conserved POU3F2 recognition site. By combining the results from these two bioinformatic tools, we obtained a set of putative target genes of POU3F2. To study the transcriptional regulation of the *NTF3* gene, the region ranging from nt − 2000 to nt 221 from the transcription start site of the *NTF3* gene was used to predict the potential transcription factors binding elements. The sequences upstream of the human *NTF*3 gene (NC_000006.11) were obtained from the National Center for Biotechnology Information (NCBI) and screened using TESS software (http://www.cbil.upenn.edu/cgi-bin/tess/tess).

### DNA Affinity Precipitation Assay

To detect the interaction between the POU3F2 protein and *Ntf3* gene, a DNA affinity precipitation assay (DAPA) was performed. Briefly, double-stranded, 5′-end-biotinylated oligonucleotides prepared by PCR were incubated with nuclear extracts from NT2D1 cells in a 100-μl reaction mixture (20 mM sodium phosphate, pH 8.0, 100 mM NaCl, 7% glycerol, 0.5 mM EDTA, 2 mM dithiothreitol, 0.1 mg/ml BSA, 0.02% Tween, and 0.02 mg/ml poly (dI-dC)) at 4 °C for 16 h. The mixtures were then incubated with 0.74% for an additional 30 min on ice to cross-link the DNA-protein complexes. The cross-linked complexes were pulled down by using M-280 Streptavidin Dynabeads (Dynal Biotech ASA, Oslo, Norway) following the manufacturer’s instructions and analyzed by Western blotting using anti-POU3F2 antibody (Abcam, Cambridge, UK). The DNA sequences for DAPA were as follows: AADC: 5′-GCTGCTCAGTAAATAATGCAGAGC-3′, and 5′-GCTCTGCA- TTATTTACTGAGCAGC-3′; NTF3: 5′-TTTCAAGGTATTTGGATTTTTTGAAC-C-3′ and 5′-GGTTCAAAAAATCCAAATACCTTGAAA-3′; NTF3_Mutant: 5′-GG-TTCAAGCGCCCGCGCACCTTGAAA-3′ and 5′-TTTCAAGGTGCGCGGGCGC-TTGTAACC-3′.

### Next-Generation-Sequence-Based POU3F2 ChIP-Sequencing

POU3F2 chromatin immunoprecipitation (ChIP) samples were prepared from NT2D1 cells harvested at the indicated times after neurogenic induction. These NT2D1 cells were fixed with 1% formaldehyde. Nuclear lysates were sonicated with a sonicator to shear the chromatin into fragments of approximately 200 bp–1 kb in size. The sonicated lysates were then incubated with anti-POU3F2 antibody (sc-6029, Santa Cruz Biotechnology, Inc., Dallas, TX) and the protein-DNA complexes precipitated with protein A/G agarose beads. After washing and reversal of the cross-link, DNA was recovered by phenol/chloroform extraction and sequenced simultaneously using the Genome Analyzer and Solexa® Sequencing technology (Illumina, Inc., San Diego, CA).

### Promoter Assay Construct and Plasmid DNA Extraction

Different lengths of *NTF3* promoter were cloned upstream of the firefly luciferase reporter separately in the pGL3-Basic vector. The NTF3_mut_pGL3-b plasmid with a mutated POU3F2 binding site (5′-ATTTTGGATT-3′ to 5′-ATGGGGAGG-3′) was provided by Protech Technology Enterprise Co, Ltd (Taipei, Taiwan).

### Plasmid Transfection and Luciferase Assay

NT2D1 cells were seeded onto 24-well culture dishes for 24 h and co-transfected with pGL3-basic promoter-driven Firefly luciferase reporter plasmid and Simian Virus 40 promoter-driven Renilla (pRL-SV40) plasmid using jetPEI (101-10, Polyplus Transfection, France) and incubated for 24 h. The activities of Firefly luciferase and Renilla luciferase were measured by using the Dual-Luciferase Reporter Assay System (Promega, Wisconsin, USA) and a GloMax™ 20/20 Luminometer. The transfection experiments and luciferase assay were performed according to the manufacturer’s instructions.

### VSV-G Pseudotyped Lentivirus-shRNA Production and Infection

The plasmids pshRNA_*POU3F2*_ (clone ID:TRCN0000230048), pshRNA_*NTF3*_ (clone ID:TRCN0000378856), and pshRNA_*Luc*_ (clone ID:TRCN0000231693) harboring shRNA targeting *POU3F2*, *NTF3*, and *luciferase* mRNAs, respectively, were purchased from the National RNAi Core Facility at Academia Sinica, Taiwan. Virus preparation and cell infection were performed as suggested by the plasmids’ provider (http://rnai.genmed.sinica.edu.tw/) and in previous studies [27, 28].

### Whole Cell Lysate Extraction and Immunoblotting

For whole cell lysate extraction, NT2D1 cells were lysed in RIPA buffer (50 mM Tris-HCl pH 7.5, 150 mM NaCl, 0.1% SDS, 0.5% sodium deoxycholate, 1% NP-40, 1× protease inhibitor cocktail) for 10 min on ice and then centrifuged at 16,000×*g* for 15 min to remove debris. Total proteins from each sample were fractionated by SDS-PAGE and then transferred to a polyvinylidene difluoride (PVDF) membrane. Monoclonal antibodies that recognize POU3F2 (#12137, Cell signaling), POU3F3 (ab90727, Abcam), NTF3 (ab65804, Abcam), β3-Tubulin (ab18207, Abcam), and GAPDH (NB300-221, NOVUS) were used to detect the expressions of POU3F2, POU3F3, NTF3, β3-Tubulin, and GAPDH, respectively. Membranes were washed for 10 min in 0.1% TBS-Tween 20, incubated in HRP-conjugated secondary antibody for 1 h, washed again, and then analyzed with the GeneGnome chemiluminescence imaging system.

### Chromatin Immunoprecipitation and Polymerase Chain Reaction

For ChIP, formaldehyde cross-linked cells were sonicated for 40 cycles (30 s on/30 s off) using a Misonix Q700 Sonicator (Qsonica, Newtown, CT) at 20% power amplitude. Briefly, 5 μg POU3F2 antibody and naive IgG were mixed with Dynabeads protein-G beads at 4 °C for 2 h. After the bead-antibody interaction, the antibody-conjugated beads were used to pull down the POU3F2-bound DNA fragments. Cross-links were reversed by incubation at 65 °C overnight. The proteins were digested by 20 μg proteinase K at 50 °C for 2 h. The DNA fragments were purified by phenol/chloroform precipitation. The DNA sequences of the NTF3 promoter occupied by POU3F2 were amplified by polymerase chain reaction (PCR). The primers used were forward 5′-GTGGGTGCAGTTCCGATG-3′ and reverse 5′-CGCTCCTCACATCATCTCCT-3′.

### Immunofluorescence

POU3F2-knockdown and control NT2D1 cells were seeded onto coverslips in six-well plates at a density of 8 × 10^4^ cells/well for the neuronal immunofluorescence assay. After exposure to neural induction medium at the indicated time points, the cells were washed in phosphate-buffered saline (PBS), fixed in 4% paraformaldehyde in PBS for 10 min at room temperature, permeabilized with 1% Triton X-100 in PBS for 10 min, and then blocked in 1% BSA in PBST for 1 h. Subsequently, cells were hybridized with anti-β3-Tubulin (ab18207, Abcam) and anti-phospho-TrkC (#NBP1-03448, Novus) antibodies overnight in blocking buffer at 4 °C. Cells were also exposed to donkey anti-rabbit-FITC antibody at room temperature for 1 h. The cells were stained with 4′, 6-diamidino-2-phenylindole (DAPI) for 5 min at room temperature and observed using a fluorescence microscope.

Mouse primary cultured neurons in a 24-well plate were fixed in 4% paraformaldehyde for 10 min. Mouse brains harvested at E18.5 or P0 were dissected and post-fixed overnight in 4% paraformaldehyde at 4 °C for 16 h. Coronal frozen sections (20 μm thick) were prepared. Primary cultured neurons and brain sections were permeabilized with 1% Triton X-100 in PBS for 10 min and then blocked in 1% bovine serum albumin in PBST for 1 h. Then, primary cultured neurons and slices were incubated overnight at 4 °C in blocking buffer containing goat anti-NTF3 antibody (#NBP1-46517, Novus) and rabbit anti-POU3F2 antibody (#12137S, Cell Signaling). Immunostaining was visualized following incubation with donkey anti-rabbit-FITC antibody at room temperature for 1 h.

### Statistical Analysis

All data are presented as mean ± SEM. The Student’s *t* test or Mann–Whitney *U* test was used for comparisons between two groups. Comparisons between more than two groups was calculated by one-way ANOVA followed by Bonferroni’s post hoc test using GraphPad Prism 5 Software (GraphPad Software, Inc. CA, USA). Results were considered statistically significant at *p* < 0.05.

## Results

### Induction of NT2D1 Neuronal Differentiation

**Fig. 1:**
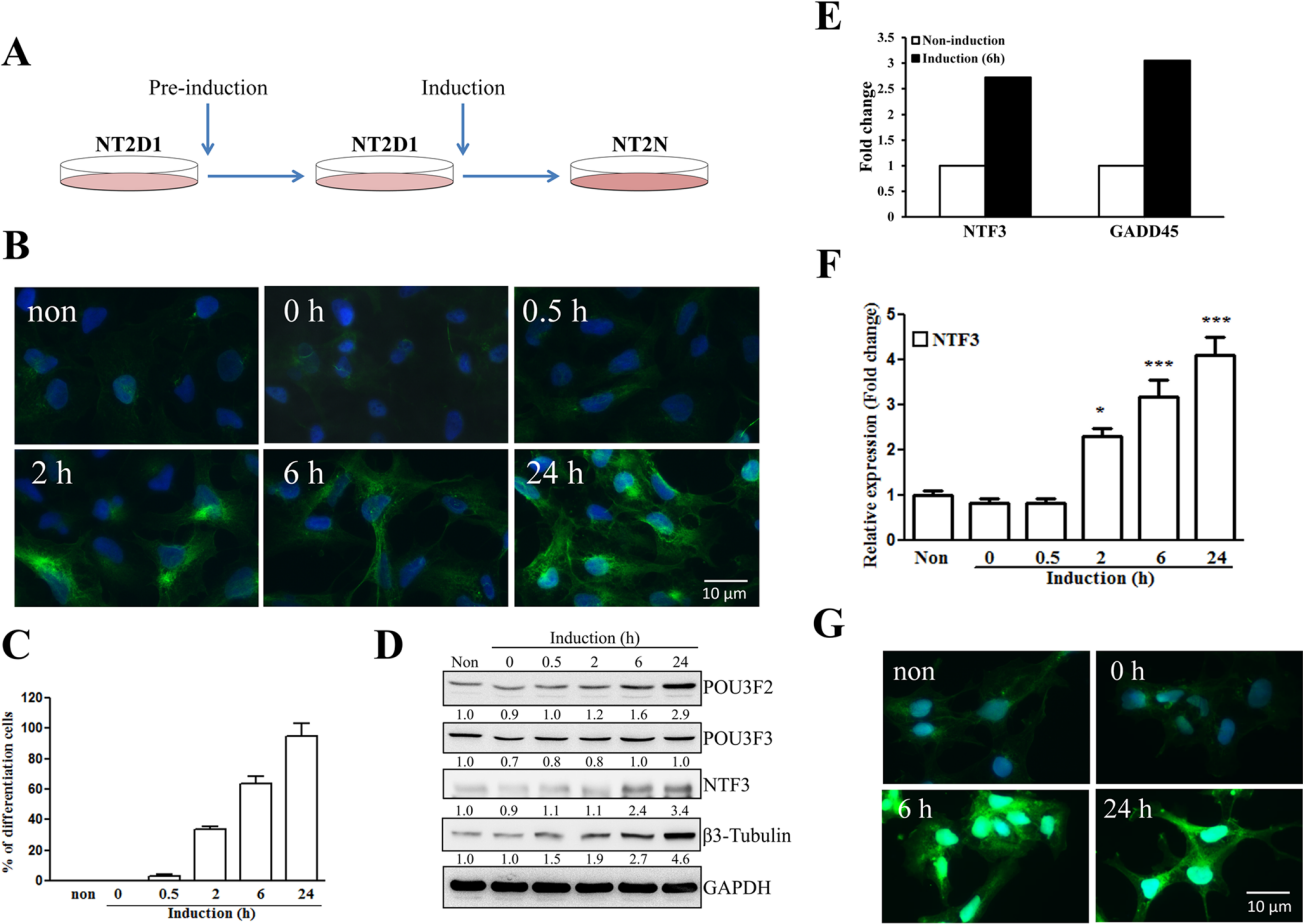
Upregulation of the transcription factors POU3F2 and NTF3 during neuronal differentiation of NT2D1. **a** The protocol for neuronal induction of NT2D1 cells is schematized. **b** β3-tubulin staining for neuronal cells in NT2D1 cells untreated (non) and treated with neuronal induction medium at the indicated time points. **c** Quantification of β3-tubulin-positive cells. **d** Immunoblotting analysis for POU3F2, POU3F3, β3-tubulin, and NTF3 in NT2D1 cells untreated or treated with neuronal induction medium at the indicated time points. The values show the expression relative to that of untreated cells (to which a value of 1 was assigned). **e** Microarray analysis showed that neuronal induction for 6 h increased the expression of NTF3 and GADD45 in NT2D1 cells. **f** *NTF3* mRNA expression after neuronal induction was analyzed by real-time PCR. The levels of mRNA were calculated as the relative expression compared with that of non-induced NT2D1 cells. GAPDH mRNA was used as a control. *p < 0.05; ***p < 0.001. **g** Phospho-TrkC (Tyr820) staining in treated and untreated NT2D1 cells. Values are presented as mean ± SEM of three independent experiments for **c** and **f**

To investigate the expression and regulation of genes in the early stages of neuronal differentiation, we treated NT2D1 cells with neuronal induction medium [23, 25, 26] following the procedure shown in Fig. 1a. Immunofluorescence staining revealed a time-dependent increase in β3-tubulin expression, a neuron-specific marker, after exposure to induction medium (Fig. 1b). As shown in Fig. 1c, the percentage of NT2D1 cells positive for β3-tubulin staining increased in a time-dependent manner, indicating induction of neuronal differentiation. The degree of β3-tubulin expression was consistent with the findings obtained by Western blot analyses (Fig. 1d). In addition, there was a significant time-dependent increase in the expression of POU3F2, but not POU3F3, during neuronal differentiation (Fig. 1d). Interestingly, neuronal induction also increased NTF3 expression (Fig. 1d). Next, we performed next-generation-sequencing-based genome-wide POU3F2 binding site screening (ChIP-seq) and bioinformatics-based mapping to identify the potential target genes of POU3F2 during the early neuronal differentiation of NT2D1 cells. A total of 26,742,029 reads were obtained from differentiated NT2D1 cells and 29,041,273 reads from undifferentiated NT2D1 cells. Analysis of ChIP-seq data identified 1175 genomic regions that were targeted by POU3F2 and revealed enriched binding peaks close to the transcription start sites (TSS) of 775 genes in early neuronal differentiation. On the other hand, in silico prediction of putative POU3F2 binding sites resulted in 8705 hits. Comparison of the in silico mapping and ChIP-seq results identified 166 putative targets of POU3F2, including *NTF3*, during early neuronal differentiation (Supplementary Table 1). An Affymetrix Human U133 Plus 2.0 oligonucleotide microarray (Affymetrix, Santa Clara, CA, USA) was also used to establish gene expression profiles of NT2D1 treated with induction medium for 6 h. Among the detected genes, *NTF3* and the known POU3F2-target gene *GADD45* were significantly overexpressed after exposure to induction medium (Fig. 1e). The results of RT-qPCR showed that *NTF3* mRNA expression was significantly upregulated in NT2D1 cells at 2, 6, and 24 h after exposure to induction medium (Fig. 1f). Simultaneously, TrkC, a receptor for NTF3, was phosphorylated after neurogenic induction, suggesting that the NTF3/TrkC signaling pathway was activated (Fig. 1g). These results prompted us to examine whether POU3F2 regulates NTF3 expression during neuronal differentiation.

### Interaction of POU3F2 with the NTF3 Gene Promoter

**Fig. 2:**
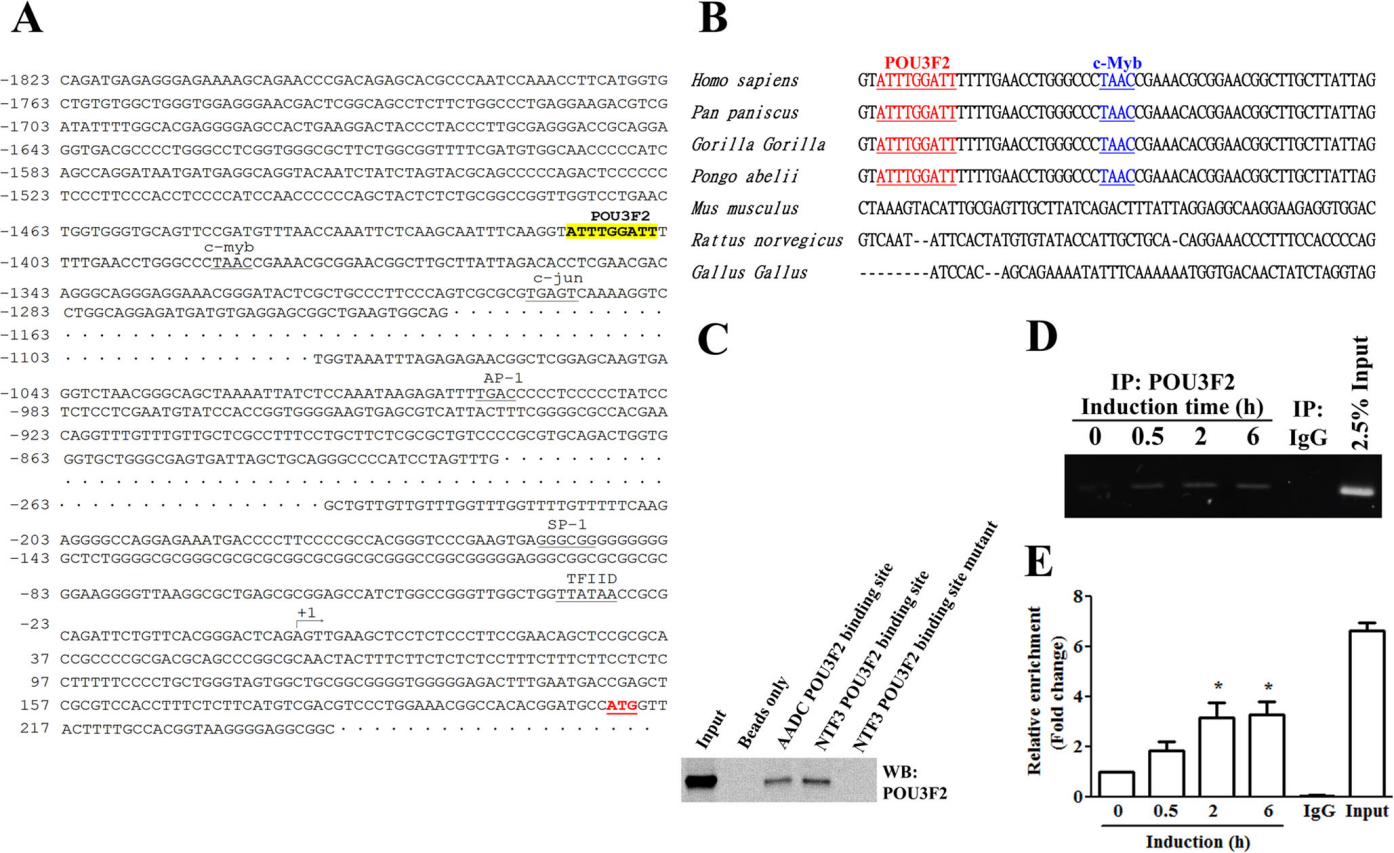
Identification of the POU3F2 binding site on the NTF3 promoter. **a** Transcription factor response elements predicted by the Transcription Element Search System for the nucleotide sequence of the *NTF3* promoter region (− 1823 to + 243). The transcription start site is indicated as + 1. **b** Comparison of NTF3 promoter sequence conservation between different species. **c** Biotin-labeled oligonucleotides containing the intact or mutated POU3F2 binding site were hybridized with total lysates prepared from NT2D1 cells. The POU3F2-DNA complexes were precipitated by streptavidin agarose beads. POU3F2 was analyzed by Western blot analyses. The input of nuclear extracts was used as loading control. Three independent experiments were performed. **d** Chromatin was prepared from NT2D1 cells treated with induction medium for 0, 2, and 6 h. Cell lysates were mixed with antibodies against POU3F2 or IgG and then precipitated. The precipitates were analyzed by PCR for the presence of the *NTF3* promoter sequence. The DNA purified from the sonicated chromatin was directly analyzed by PCR using the ChIP primer, which was used as an input control (Input). **e** The values of the ChIP DNA were normalized to that of the NT2D1 cells at 0 h (as a control). Values of fold-change over the control are presented as mean ± SEM of three independent experiments for **d**. *p < 0.05 compared with the control

We further applied bioinformatic tools to analyze the promoter region of the *NTF3* gene, an approximately 1.8-kb DNA sequence upstream of the transcription start site, for the prediction of transcriptional regulatory elements. We found a potential POU3F2 response element (nt − 1413 to nt − 1405) in the *NTF3* promoter (Fig. 2a) using the TESS, suggesting *NTF3* as a novel target gene of POU3F2. The flanking sequence of the predicted POU3F2 binding site in the *NTF3* promoter was conserved in humans, bonobos, gorillas, and Sumatran orangutans but not in mice, rats, or chickens (Fig. 2b). To confirm the putative binding of POU3F2 to the *NTF3* promoter, we performed DAPA in NT2D1 cells. As shown in Fig. 2c, POU3F2 was pulled down by the specific probes derived from the promoter of *NTF3* and *aromatic L*-*amino acid decarboxylase* (*AADC*, a target gene of POU3F2), but not by the *NTF3* probe with the POU3F2 binding site mutated. To investigate the binding ability of POU3F2 to the *NTF3* promoter during neuronal differentiation, a ChIP assay was performed in NT2D1 cells exposed to induction medium. The association between POU3F2 and the *NTF3* promoter was markedly enhanced after 0.5, 2, and 6 h of exposure to induction medium, compared with the baseline control (Fig. 2d, e). These results suggested that POU3F2 bound to the *NTF3* promoter.

### POU3F2 Transactivated the Promoter of NTF3 Gene

**Fig. 3:**
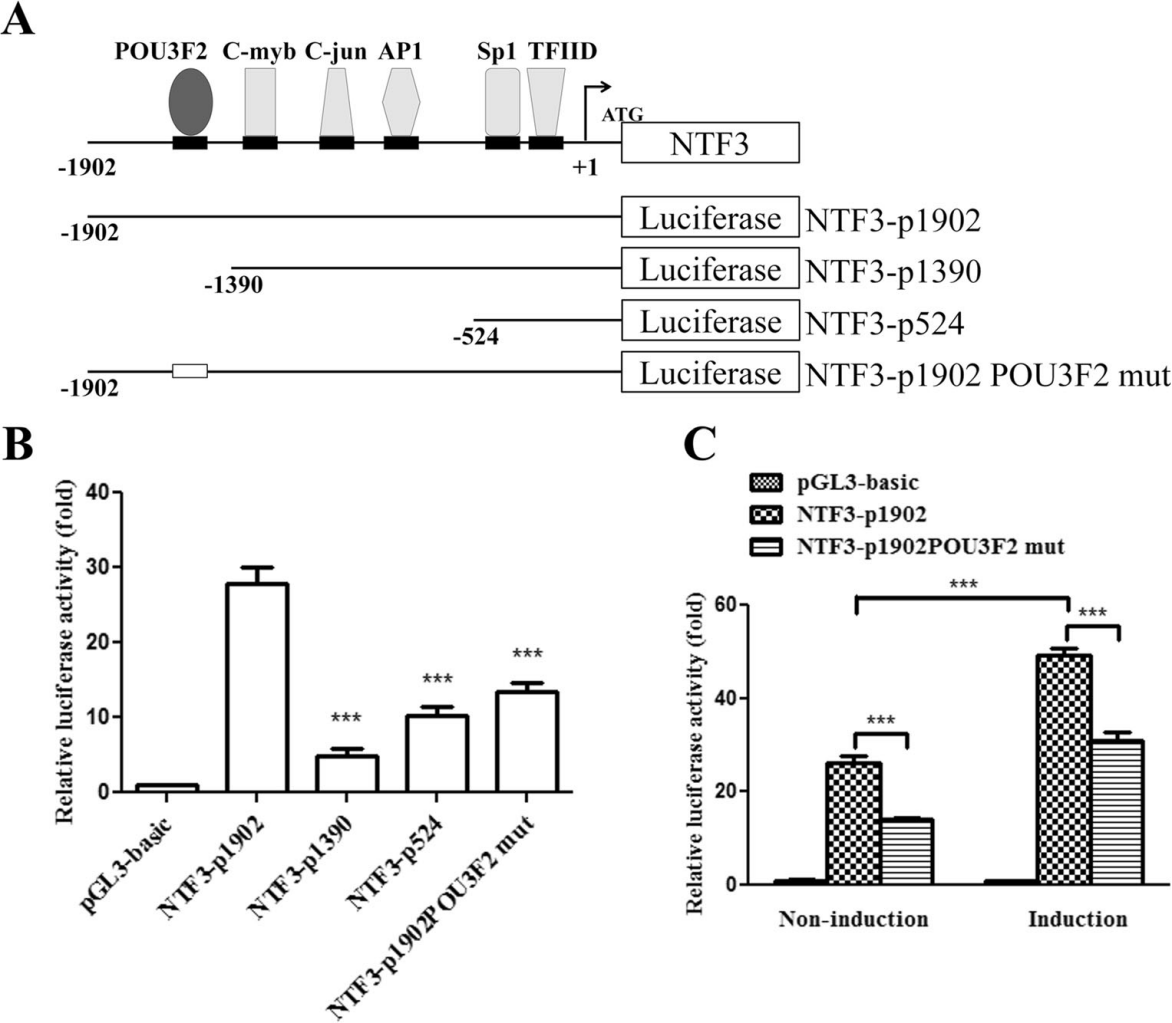
Effects of POU3F2 on NTF3 promoter activity. **a** Schematic representation of NTF3-luciferase chimeric constructs. The negative numbers refer to the numbers of bases upstream of the transcription start (+ 1) site of the *NTF3* gene. **b** NT2D1 cells were transiently transfected with the pGL3 basic vector or *NTF3* promoter constructs of different lengths. The luciferase activity of each reporter was normalized to the Renilla luciferase activity and compared with that of cells transfected with the pGL3 basic vector (to which a value of 1 was assigned). ***p < 0.001. **c** NT2D1 cells were transfected with the pGL3 basic vector, pNTF3-1902, and pNTF3-1902 POU3F2 mut. Approximately 24 h later, cells were treated with neuronal induction medium. The transcriptional activity of each reporter was normalized to the Renilla luciferase activity and compared with that of cells transfected with the pGL3 basic vector (to which a value of 1 was assigned). ***p < 0.001. Values are presented as mean ± SEM of three independent experiments for **b** and **c**

Next, we performed promoter truncation and reporter assays to examine if POU3F2 transactivated the promoter of the *NTF3* gene. The luciferase reporter constructs containing regions of the NTF3 promoter varying in length are shown in Fig. 3a. We transfected these constructs individually into NT2D1 cells and found that the luciferase activities of cells receiving NTF3-p1390 and NTF3-p524 were significantly lower than that of cells receiving NTF3-p1902 (Fig. 3b). Moreover, the luciferase activity of cells transfected with the POU3F2 binding site-mutated NTF3-p1902 (designated as NTF3-p1902 POU3F2 mut) was approximately 50% of that of cells receiving NTF3-p1902 (Fig. 3b). Next, we transfected cells with NTF3-p1902 or NTF3-p1902 POU3F2 mut, and induced cells to undergo neuronal differentiation. As shown in Fig. 3c, the luciferase activity of cells receiving either construct was significantly increased by neuronal induction, compared with non-induction controls. However, an approximately 40% loss of luciferase activity was observed in the cells receiving NTF3-p1902 POU3F2 mut, compared with that in cells receiving NTF3-p1902. These data demonstrated that the POU3F2 binding element in the *NTF3* promoter was important for its activation by neuronal induction and that POU3F2 was able to transactivate *NTF3* promoter activity in NT2D1 cells during neuronal differentiation.

### POU3F2 Silencing Diminished NTF3 Expression

**Fig. 4:**
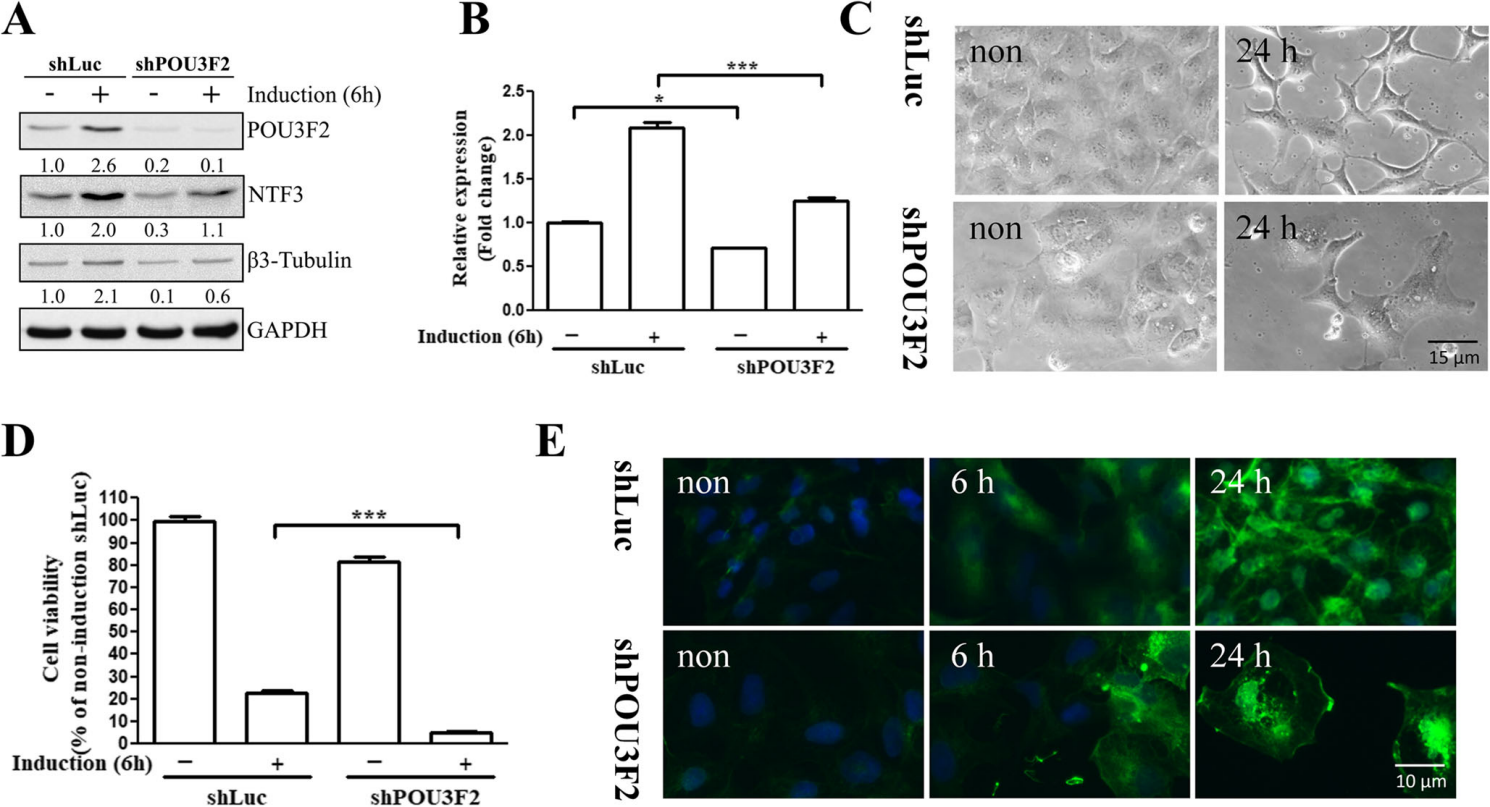
Effects of POU3F2 silencing on neuronal differentiation and NTF3 expression in NT2D1 cells. **a** POU3F2 expression in POU3F2-knockdown (shPOU3F2) and control (shLuc) NT2D1 cells was determined by Western blot analyses after neuronal induction for 6 h. GAPDH was used as a loading control. The values represent the relative expression compared with that of the non-induced shLuc cells (to which a value of 1 was assigned). **b** NTF3 mRNA expression of the cells described in **a** was analyzed by real-time PCR. mRNA levels were calculated relative to that of the non-induced shLuc cells. *p < 0.05; ***p < 0.001. **c** Neuronal morphology of shLuc and shPOU3F2 cells that were treated with neuronal induction medium for 24 h or left untreated (non). **d** Quantification of cell numbers of shLuc and shPOU3F2 described in **c**. All the percentages of the shLuc and shNTF3 cells were compared to that of the non-induction shLuc cells (to which a value of 100% was assigned). **e** β3-tubulin staining was performed on shLuc and shPOU3F2 cells, which were treated with neuronal induction medium for 0, 6, or 24 h or left untreated, after which neuronal cells were detected. Values represent the mean ± SEM of three independent experiments for **b** and **d**

To further address the regulatory role of POU3F2 in NTF3 expression, we knocked down POU3F2 expression in NT2D1 cells with a VSV-G pseudotyped lentivirus-shRNA system and then examined the NTF3 expression. As shown in Fig. 4a, POU3F2 knockdown decreased POU3F2 and NTF3 expression in NT2D1 cells with or without neuronal induction. *NTF3* mRNA expression was also significantly inhibited in the POU3F2-knockdown NT2D1 cells, with or without neuronal induction (Fig. 4b). Moreover, we examined the impact of POU3F2 knockdown on neuronal differentiation. We found that the viability of POU3F2-knockdown cells was approximately 80% of that of control cells before neuronal induction and was markedly lowered to approximately one fourth of that of corresponding control cells after neuronal induction for 24 h (Fig. 4c, d). Immunostaining showed that viable POU3F2-knockdown cells expressed β3-tubulin 6 and 24 h after neuronal induction. However, POU3F2-knockdown cells appeared to diminish the dendritic morphology seen in the control cells (Fig. 4e). These results suggested that POU3F2 played a regulatory role in NTF3 expression and had a critical effect on cell viability during neuronal differentiation.

### Effects of Recombinant NTF3 Protein on the Neuronal Differentiation of NTF3-Silencing and POU3F2-Silencing NT2D1 Cells

**Fig. 5:**
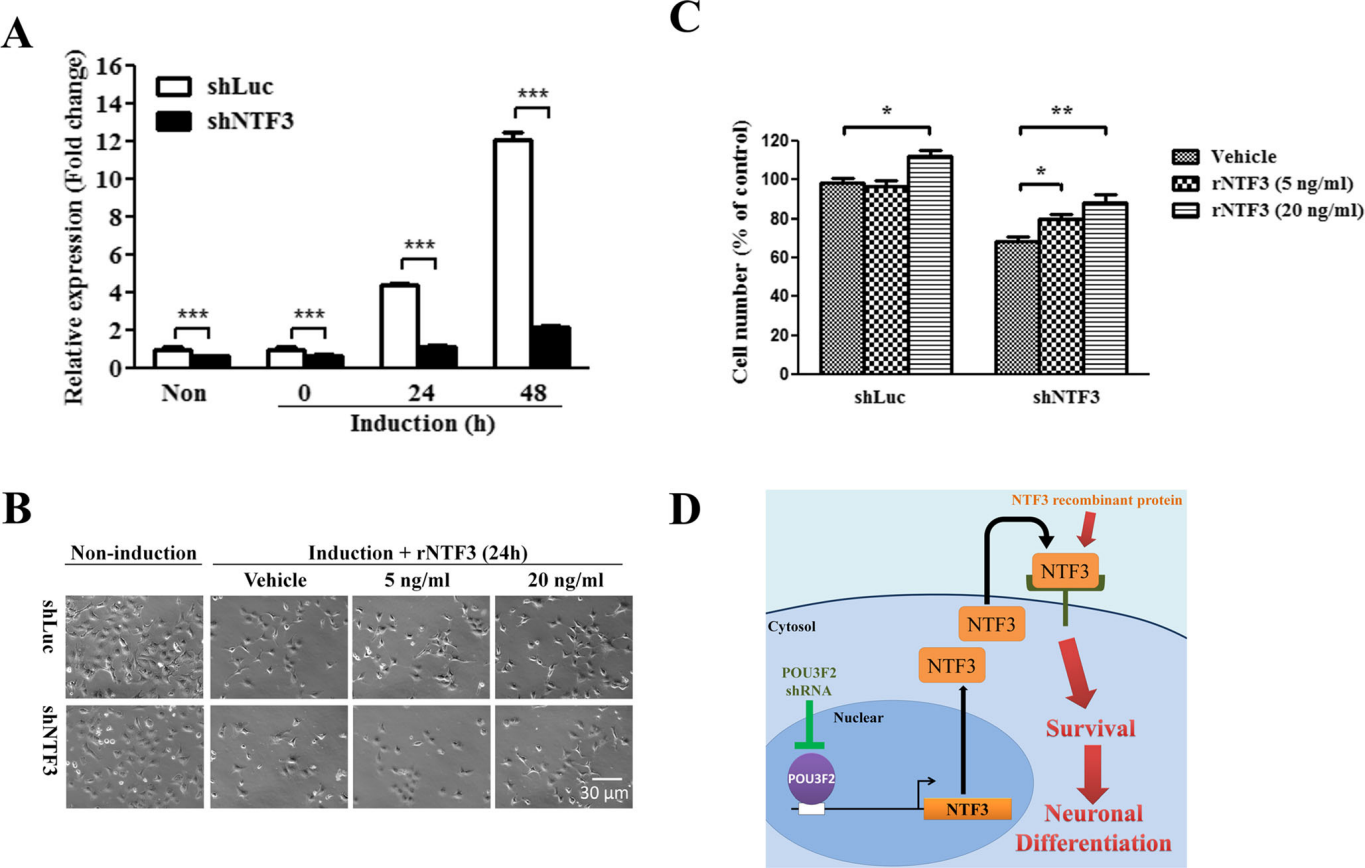
Effects of NTF3 silencing and NTF3 recombinant protein treatment on the viability and neuronal differentiation of NT2D1 cells. **a** *NTF3* mRNA levels in NTF3-knockdown (shNTF3) and control (shLuc) NT2D1 cells, which were treated with neuronal induction medium for 0, 24, or 48 h or left untreated (Non), were determined by real-time PCR. mRNA levels were calculated as the relative expression compared with the untreated shLuc cells. ***p < 0.001. **b** Phase contrast microscopy images of untreated shLuc and shNTF3 cells and those cells 24 h after neuronal induction with concomitant treatment of rNTF3 (5, 20 ng/ml) or vehicle. **c** Quantification of neuron number of shLuc and shNTF3 cells as described in **b**. All the percentages of neurons differentiated from shLuc and shNTF3 cells were compared to that of neurons differentiated from the vehicle-treated shLuc cells (to which a value of 100% was assigned). *p < 0.05; **p < 0.01. **d** A suggested model of the POU3F2/NTF3 pathway that mediates the process of neuron differentiation. Values are presented as mean ± SEM of at least three independent experiments for **a** and **c**

Next, to evaluate the role of NTF3 in POU3F2-based neuronal differentiation, we knocked down NTF3 expression in NT2D1 cells for subsequent examinations. As shown in Fig. 5a, while neuronal induction increased NTF3 expression time-dependently in control cells, NTF3 knockdown significantly decreased the expression of *NTF3* mRNA in the untreated cells and inhibited the induction of *NTF3* mRNA expression during neuronal differentiation. After neuronal induction for 24 h, the number of neurons differentiated from the vehicle-treated NTF3-knockdown cells was significantly lowered to 70% of that from the vehicle-treated control cells (Fig. 5b, c). Administration of 5 ng/ml recombinant NTF3 (rNTF3) did not increase the number of differentiated neurons from control cells but caused an approximately 14.3% increase in the number of differentiated neurons from NTF3-knockdown cells. At a concentration of 20 ng/ml, rNTF3 caused an approximately 15 and 28.5% increase in the differentiated neurons from control cells and NTF3-knockdown cells, respectively (Fig. 5c). We also examined the role of NTF3 in the differentiation of POU3F2-knockdown cells and found that 20 ng/ml rNTF3 significantly increased the number of control and POU3F2-knockdown cells that differentiated into neurons (Supplementary Fig. 1). These results indicated that NTF3 plays an important role in the neuronal differentiation of NT2D1 cells and that the POU3F2/NTF3 pathway is involved in the process of neuron differentiation (Fig. 5d).

### POU3F2 and NTF3 Proteins Were Colocalized in the Developing Mouse Neurons In Vitro and In Vivo

**Fig. 6:**
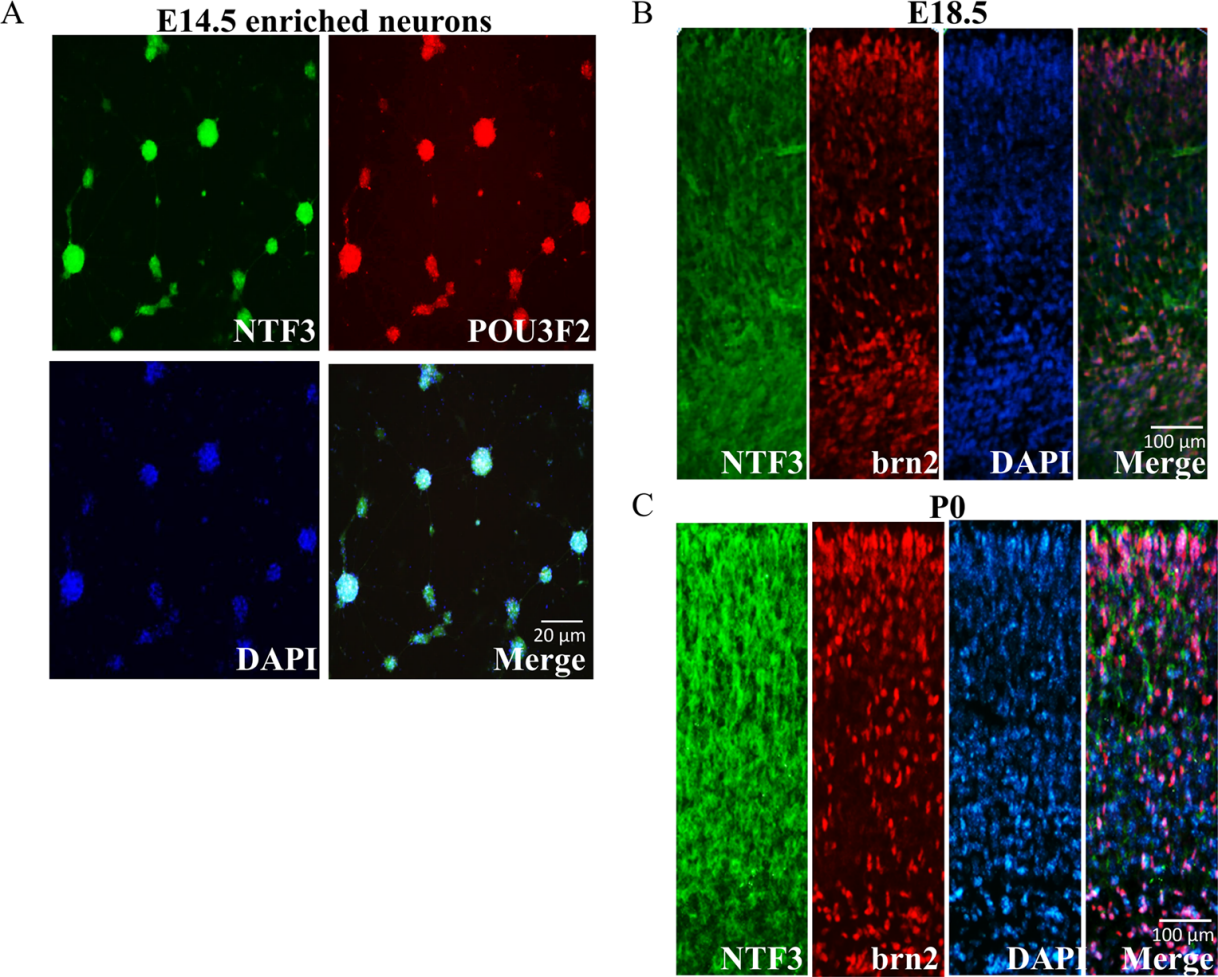
Colocalization of POU3F2 and NTF3 proteins in the developing mouse neurons. **a** Primary mouse neurons (EN) were prepared from E14.5 embryos, cultured for 7 days, and subjected to double fluorescence immunostaining using anti-NTF3 (green) and anti-POU3F2 (red) antibodies and FITC. Immunostaining was also performed on cortical sections of mouse brain prepared from E18.5 embryos (**b**) and postnatal day (P) 0 mice (**c**). Merged images of NTF3 and POU3F2 showed extensive overlap of expression in primary enriched cultured neurons and in the E18.5 and P0 cortex, mainly at the upper and deeper layers

Given the important role that the POU3F2/NTF3 pathway plays in the neuronal differentiation of human NT2D1 cells, although the flanking sequence of the predicted POU3F2 binding site in human *NTF3* promoter is not conserved in mice, rats, and chickens, we thought that this pathway might be also involved in neuronal differentiation in these three species. Therefore, we performed bioinformatic prediction on the *Ntf3* promoter sequences of these three species and found putative POU3F2 binding sites (mouse: nt − 1314 to nt − 1308, nt − 1206 to nt − 1200; rat: nt − 1110 to nt − 1116, nt − 825 to nt − 831; chicken: nt − 814 to nt − 808). We subsequently prepared primary enriched neurons from mice at embryonic day (E) 14.5, and brain sections from mice at E18.5 and postnatal day (P) 0. Double immunostaining using anti-NTF3 (green) and anti-POU3F2 (red) antibodies on the enriched neurons and brain sections showed extensive overlap in the expression of POU3F2 and NTF3 (Fig. 6a–c). These in vivo and in vitro data indicated colocalization of POU3F2 and NTF3 in developing neurons and suggested that the function of POU3F2/NTF3 pathway in neuronal differentiation is conserved in humans and mice.

## Discussion

POU3F2 expression has been shown to be induced in embryonic stem cell-derived progenitor cells, suggesting that POU3F2 plays an important role in neuronal differentiation [7]. However, little is known about the genes it regulates to mediate neurogenesis. Here, we provide several lines of evidence to show that NTF3 is an important target of POU3F2 in regulating human neuronal differentiation. Our data showed that (i) POU3F2 binds to and transactivates *NTF3* in NT2D1 cells with or without neuronal induction (Figs. 2 and 3), (ii) the interaction between POU3F2 and the *NTF3* promoter is increased during neuronal differentiation (Fig. 2d), (iii) POU3F2 knockdown decreases NTF3 expression (Fig. 4), and (iv) NTF3 knockdown decreases the number of differentiated neurons, which can be partially recovered by rNTF3 (Fig. 5). These results depicted a regulatory role of POU3F2 in NTF3 expression and suggested that POU3F2 might maintain the basal expression of NTF3 in neuroprogenitor cells and further increase NTF3 expression in response to neuronal induction.

In fact, given our finding that rNTF3 (20 ng/ml) increased the number of differentiated neurons from POU3F2-knockdown cells (Supplementary Fig. 1), NTF3 could be an important downstream mediator of POU3F2 in promoting human neuronal differentiation. It is worthy of note that the outgrowth dendrite of neurons differentiated from POU3F2-knockdown NT2D1 cells was significantly abolished compared with those from control cells (Fig. 4c, e). These results indicate that POU3F2 also plays a role in the development of neuronal morphology.

On the other hand, although the flanking sequence of the predicted POU3F2 binding site in the human *NTF3* promoter was not conserved in mice, rats, and chickens (Fig. 2b), bioinformatics prediction showed the existence of putative POU3F2 binding sites in the *Ntf3* promoter sequences of these three species. Indeed, our data demonstrated the colocalization and coexpression of POU3F2 and NTF3 in mouse primary neuron cultures and in the brain tissues of mice at E18.5 and P0 (Fig. 6), which also supported a regulatory role of the POU3F2/NTF3 pathway in mouse neuronal development.

Our results showed that the promoter activity of NTF3-p1390 and NTF3-p1902 POU3F2 mut were approximately 80 and 50% lower, respectively, than that of NTF3-p1902 (Fig. 3), indicating that the protein-DNA interactions taking place in the region from nt − 1902 to nt − 1930 of the *NTF3* promoter were important contributors to its transactivation. Using transcription factor prediction software, we found a putative SRY response element and several putative Sp-family response elements in the human *NTF3* promoter ranging from nt − 1920 to nt − 1390. It has been reported that Sp-family transcription factors and SRY positively regulate the *Ntf3* promoter in mouse neuron and rat testis, respectively [29, 30]. Furthermore, whereas the promoter activity of NTF3-p524 was approximately 50% higher than that of NTF3-p1390 (Fig. 3), sequence analysis of the *NTF3* promoter region ranging from nt − 1390 to nt − 524 also revealed several putative binding sites for some important differentiation-related transcription factors such as CTCF, YY1, GATA-3, and Sp-1 [31]. In addition, the regulatory role of Zic 1 in neuronal differentiation has been addressed in embryonic mouse models [16]. We found that POU3F2 knockdown resulted in decreased Zic1 expression in NT2D1 cells with or without neuronal induction (Supplementary Fig. 2). We also found a putative Zic1 binding site in the human *NTF3* promoter via bioinformatics prediction. Therefore, POU3F2 may also regulate NTF3 expression via the POU3F2/Zic 1 axis in developing human neurons. While our data, taken together, depict POU3F2 as a transactivator of the *NTF3* gene during neuronal differentiation, it is possible that POU3F2 may target other factors for NTF3 induction and that transcription factors other than POU3F2 may be also involved in NTF3 induction. More studies are needed to clarify the transcriptional network which regulates *NTF3* promoter in response to neurogenic induction.

To the best of our knowledge, this is the first study to reveal a novel function of POU3F2 in regulating the promoter activity and expression of NTF3. Furthermore, we found that NTF3 promotes the survival/differentiation cascade during neuronal differentiation. Our findings provide further evidence that human neuronal differentiation and survival is partially governed by the POU3F2/NTF3 pathway.

## Electronic Supplementary Material

Supplementary Table 1 (DOCX 30 kb).

Supplementary Fig. 1 (GIF 103 kb).

High Resolution Image (TIFF 16170 kb).

Supplementary Fig. 2 (GIF 67 kb).

High Resolution Image (TIFF 16427 kb).
