# Supplementary material for: NTF3 Is a Novel Target Gene of the Transcription Factor POU3F2 and Is Required for Neuronal Differentiation
Source: Mol Neurobiol. 2018 Mar 16;55(11):8403–13. doi: 10.1007/s12035-018-0995-y (PMC6153716; doi:10.1007/s12035-018-0995-y)
Supplement: Supplementary file 1 — (DOCX 30 kb). [file 12035_2018_995_MOESM1_ESM.docx]

**Supplementary Table 1.** A list of putative POU3F2-target genes, identified by both in silico mapping and ChIP-seq during early neuronal differentiation. The gene symbol and reference sequence (RefSeq) ID are reported

| **Gene Symbol** | | **RefSeq ID** | | **Gene Symbol** | | **RefSeq ID** | | | **Gene Symbol** | | **RefSeq ID** | | |  |  |
| --- | --- | --- | --- | --- | --- | --- | --- | --- | --- | --- | --- | --- | --- | --- | --- |
| ABLIM2 | | NM_001130085 | | CACNA1C | | NM_001129836 | | | CORO1B | | NM_020441 | | |  |  |
| ADAMTS4 | | NM_005099 | | CACNA1C | | NM_001129833 | | | CRHR2 | | NM_001202482 | | |  |  |
| AGPAT3 | | NM_001037553 | | CACNA1C | | NM_001129839 | | | CRHR2 | | NM_001202481 | | |  |  |
| AGT | | NM_000029 | | CACNA1C | | NM_001129834 | | | CRHR2 | | NM_001202475 | | |  |  |
| AHDC1 | | NM_001029882 | | CACNA1C | | NM_001129832 | | | DAB2IP | | NM_032552 | | |  |  |
| ALX3 | | NM_006492 | | CACNA1C | | NM_001129841 | | | DALRD3 | | NM_001009996 | | |  |  |
| AMN1 | | NM_001113402 | | CACNA1C | | NM_001129829 | | | DALRD3 | | NM_018114 | | |  |  |
| ANO2 | | NM_020373 | | CACNA1C | | NM_001129837 | | | DGKZ | | NM_003646 | | |  |  |
| AP2M1 | | NM_001025205 | | CACNA1C | | NM_001167625 | | | DGKZ | | NM_001105540 | | |  |  |
| AP2M1 | | NM_004068 | | CACNA1C | | NM_199460 | | | DGKZ | | NM_201532 | | |  |  |
| APOA1BP | | NM_144772 | | CACNA1C | | NM_001129843 | | | DNAJB8 | | NM_153330 | | |  |  |
| APOA2 | | NM_001643 | | CACNA1C | | NM_001167624 | | | DOK7 | | NM_001164673 | | |  |  |
| ARHGAP30 | | NM_181720 | | CACNA1C | | NM_001129830 | | | DOK7 | | NM_173660 | | |  |  |
| ATF3 | | NM_001206486 | | CACNA1C | | NM_001129827 | | | DPH2 | | NM_001039589 | | |  |  |
| ATP1A2 | | NM_000702 | | CACNA1C | | NM_001129842 | | | DPH2 | | NM_001384 | | |  |  |
| BCAT1 | | NM_001178094 | | CACNA1C | | NM_001129838 | | | DYRK4 | | NM_003845 | | |  |  |
| BCL2L14 | | NM_138722 | | CASQ1 | | NM_001231 | | | EFCAB4B | | NM_001144958 | | |  |  |
| BCL2L14 | | NM_030766 | | CATSPER4 | | NM_198137 | | | EFCAB4B | | NM_032680 | | |  |  |
| BCL2L14 | | NM_138723 | | CCDC28B | | NM_024296 | | | EHD1 | | NM_006795 | | |  |  |
| C12orf35 | | NM_018169 | | CDC20 | | NM_001255 | | | EMG1 | | NM_006331 | | |  |  |
| C1orf122 | | NM_001142726 | | CDC42EP2 | | NM_006779 | | | EPB41 | | NM_001166005 | | |  |  |
| C1orf204 | | NM_001134233 | | CDCP2 | | NM_201546 | | | EXTL1 | | NM_004455 | | |  |  |
| C1orf35 | | NM_024319 | | CDK5 | | NM_004935 | | | FAM3B | | NM_058186 | | |  |  |
| C9orf114 | | NM_016390 | | CDK5 | | NM_001164410 | | | FAM63A | | NM_001040217 | | |  |  |
| CA14 | | NM_012113 | | CHI3L1 | | NM_001276 | | | FBXL14 | | NM_152441 | | |  |  |
| CACNA1C | | NM_001129844 | | CHIT1 | | NM_003465 | | | FDPS | | NM_001135821 | | |  |  |
| CACNA1C | | NM_001129831 | | CISH | | NM_013324 | | | FGD4 | | NM_139241 | | |  |  |
| CACNA1C | | NM_001167623 | | CLK3 | | NM_001130028 | | | FHL3 | | NM_004468 | | |  |  |
| CACNA1C | | NM_001129835 | | CLSTN3 | | NM_014718 | | | GCDH | | NM_000159 | | |  |  |
| CACNA1C | | NM_001129840 | | CNTN2 | | NM_005076 | | | GCDH | | NM_013976 | | |  |  |
| CACNA1C | | NM_001129846 | | COMP | | NM_000095 | | | GLI2 | | NM_005270 | | |  |  |
| CACNA1C | | NM_000719 | | CORO1B | | NM_001018070 | | | GPRC5A | | NM_003979 | | |  |  |
| **Gene Symbol** | | **RefSeq ID** | | **Gene Symbol** | | | **RefSeq ID** | | | **Gene Symbol** | | | **RefSeq ID** | | |
| GPRC5D | | NM_018654 | | LRRC23 | | | NM_001135217 | | | PDE4DIP | | | NM_001198832 | | |
| GPRIN2 | | NM_014696 | | LRRC23 | | | NM_006992 | | | PDE4DIP | | | NM_001198834 | | |
| GRHL3 | | NM_021180 | | LRRC23 | | | NM_201650 | | | PDE9A | | | NM_002606 | | |
| GRHL3 | | NM_198174 | | M6PR | | | NM_001207024 | | | PDE9A | | | NM_001001585 | | |
| GRHL3 | | NM_198173 | | MAN1C1 | | | NM_020379 | | | PDE9A | | | NM_001001584 | | |
| GSG1 | | NM_001206842 | | MCF2L | | | NM_001112732 | | | PDE9A | | | NM_001001583 | | |
| HCRTR1 | | NM_001525 | | MCF2L | | | NM_024979 | | | PDE9A | | | NM_001001582 | | |
| HEYL | | NM_014571 | | MED22 | | | NM_133640 | | | PDE9A | | | NM_001001581 | | |
| HHATL | | NM_020707 | | MED22 | | | NM_181491 | | | PDE9A | | | NM_001001580 | | |
| IFFO1 | | NM_001039670 | | MKNK1 | | | NM_003684 | | | PDE9A | | | NM_001001579 | | |
| IFFO1 | | NM_001193457 | | MLF2 | | | NM_005439 | | | PDE9A | | | NM_001001578 | | |
| IFFO1 | | NM_080730 | | MRPL24 | | | NM_145729 | | | PDE9A | | | NM_001001577 | | |
| IGFN1 | | NM_001164586 | | MRPL49 | | | NM_004927 | | | PDE9A | | | NM_001001576 | | |
| IL16 | | NM_004513 | | MRPL51 | | | NM_016497 | | | PDE9A | | | NM_001001575 | | |
| ING4 | | NM_001127586 | | MTMR11 | | | NM_181873 | | | PDE9A | | | NM_001001574 | | |
| ING4 | | NM_016162 | | MYEOV | | | NM_138768 | | | PDE9A | | | NM_001001573 | | |
| ING4 | | NM_001127582 | | MYL7 | | | NM_021223 | | | PDE9A | | | NM_001001572 | | |
| ING4 | | NM_001127585 | | MYOM3 | | | NM_152372 | | | PDE9A | | | NM_001001571 | | |
| ING4 | | NM_001127584 | | NCAPD2 | | | NM_014865 | | | PDE9A | | | NM_001001570 | | |
| ING4 | | NM_001127583 | | NDUFS2 | | | NM_001166159 | | | PDE9A | | | NM_001001569 | | |
| IPO13 | | NM_014652 | | NDUFS2 | | | NM_004550 | | | PDE9A | | | NM_001001568 | | |
| KCNA1 | | NM_000217 | | NES | | | NM_006617 | | | PDE9A | | | NM_001001567 | | |
| KCNA5 | | NM_002234 | | NLE1 | | | NM_018096 | | | PEA15 | | | NM_003768 | | |
| KCNJ9 | | NM_004983 | | NLE1 | | | NM_001014445 | | | PGP | | | NM_001042371 | | |
| KIAA1467 | | NM_020853 | | NRIP2 | | | NM_031474 | | | PHC2 | | | NM_198040 | | |
| KIAA1522 | | NM_001198973 | | NTF3 | | | NM_002527 | | | PIGO | | | NM_152850 | | |
| KRT80 | | NM_001081492 | | NTF3 | | | NM_001102654 | | | PIGO | | | NM_001201484 | | |
| KRT80 | | NM_182507 | | NUAK2 | | | NM_030952 | | | PIGO | | | NM_032634 | | |
| LDLRAD1 | | NM_001010978 | | OSBPL2 | | | NM_014835 | | | PPFIA4 | | | NM_015053 | | |
| LFNG | | NM_001166355 | | OSBPL2 | | | NM_144498 | | | PRDM15 | | | NM_001040424 | | |
| LFNG | | NM_001040168 | | PAQR6 | | | NM_024897 | | | PRDM15 | | | NM_022115 | | |
| LFNG | | NM_001040167 | | PAQR6 | | | NM_198406 | | | PRR4 | | | NM_007244 | | |
| LFNG | | NM_002304 | | PARS2 | | | NM_152268 | | | PTPN6 | | | NM_080548 | | |
| LMNA | | NM_005572 | | PCSK9 | | | NM_174936 | | | PTPN6 | | | NM_002831 | | |
| LOC653486 | | NM_001097610 | | PDCD1 | | | NM_005018 | | | PTPN6 | | | NM_080549 | | |
| **Gene Symbol** | **RefSeq ID** | | **Gene Symbol** | | **RefSeq ID** | | | **Gene Symbol** | | | | **RefSeq ID** | | |  |
| PTPRU | NM_001195001 | | SLC27A3 | | NM_024330 | | | VEGFB | | | | NM_003377 | | |  |
| PTPRU | NM_133177 | | SLC32A1 | | NM_080552 | | | YIF1A | | | | NM_020470 | | |  |
| PTPRU | NM_005704 | | SLC6A12 | | NM_001206931 | | | YRDC | | | | NM_024640 | | |  |
| PTPRU | NM_133178 | | SLC6A12 | | NM_001122848 | | | ZC3H12A | | | | NM_025079 | | |  |
| PYGO2 | NM_138300 | | SLC6A12 | | NM_001122847 | | | ZNF496 | | | | NM_032752 | | |  |
| RAB3IL1 | NM_013401 | | SLC6A12 | | NM_003044 | | | ZNF595 | | | | NM_182524 | | |  |
| RALGDS | NM_006266 | | SLC6A17 | | NM_001010898 | | |  | | | |  | | |  |
| RIIAD1 | NM_001144956 | | SLC6A9 | | NM_201649 | | |  | | | |  | | |  |
| RIMS3 | NM_014747 | | SLC6A9 | | NM_006934 | | |  | | | |  | | |  |
| RIPK4 | NM_020639 | | SLC6A9 | | NM_001024845 | | |  | | | |  | | |  |
| RPL7A | NM_000972 | | SSBP3 | | NM_001009955 | | |  | | | |  | | |  |
| RPS6KA1 | NM_001006665 | | STXBP2 | | NM_001127396 | | |  | | | |  | | |  |
| RRP1 | NM_003683 | | STXBP2 | | NM_006949 | | |  | | | |  | | |  |
| RSPO1 | NM_001038633 | | SURF2 | | NM_017503 | | |  | | | |  | | |  |
| RSPO1 | NM_001242908 | | SYT12 | | NM_001177880 | | |  | | | |  | | |  |
| RSPO1 | NM_001242910 | | SYT12 | | NM_177963 | | |  | | | |  | | |  |
| RSPO1 | NM_001242909 | | TAGLN2 | | NM_003564 | | |  | | | |  | | |  |
| S100A5 | NM_002962 | | TCIRG1 | | NM_006053 | | |  | | | |  | | |  |
| SCGB1C1 | NM_145651 | | THEM5 | | NM_182578 | | |  | | | |  | | |  |
| SCNN1A | NM_001159575 | | TMEM54 | | NM_033504 | | |  | | | |  | | |  |
| SCNN1A | NM_001159576 | | TMEM79 | | NM_032323 | | |  | | | |  | | |  |
| SCNN1A | NM_001038 | | TMTC1 | | NM_175861 | | |  | | | |  | | |  |
| SDCCAG3 | NM_006643 | | TNNI1 | | NM_003281 | | |  | | | |  | | |  |
| SDCCAG3 | NM_001039707 | | TOE1 | | NM_025077 | | |  | | | |  | | |  |
| SDCCAG3 | NM_001039708 | | TOMM40L | | NM_032174 | | |  | | | |  | | |  |
| SERINC2 | NM_018565 | | TRIM62 | | NM_018207 | | |  | | | |  | | |  |
| SERPINH1 | NM_001207014 | | TTC22 | | NM_001114108 | | |  | | | |  | | |  |
| SH2D2A | NM_001161441 | | TTC22 | | NM_017904 | | |  | | | |  | | |  |
| SH2D2A | NM_001161442 | | UBQLN4 | | NM_020131 | | |  | | | |  | | |  |
| SH2D2A | NM_003975 | | UMODL1 | | NM_001199528 | | |  | | | |  | | |  |
| SH2D2A | NM_001161444 | | UMODL1 | | NM_001199527 | | |  | | | |  | | |  |
| SH2D2A | NM_001161443 | | UMODL1 | | NM_173568 | | |  | | | |  | | |  |
| SLC26A9 | NM_134325 | | UMODL1 | | NM_001004416 | | |  | | | |  | | |  |
| SLC26A9 | NM_052934 | | USF1 | | NM_207005 | | |  | | | |  | | |  |
