# Supplementary figures and images for: NTF3 Is a Novel Target Gene of the Transcription Factor POU3F2 and Is Required for Neuronal Differentiation
Source: Mol Neurobiol. 2018 Mar 16;55(11):8403–13. doi: 10.1007/s12035-018-0995-y (PMC6153716; doi:10.1007/s12035-018-0995-y)

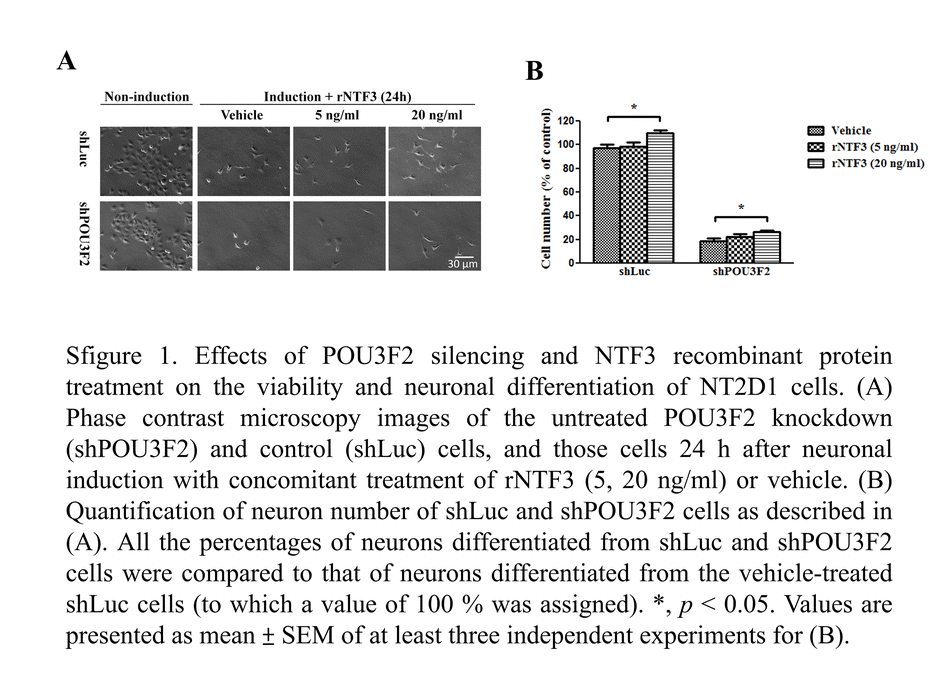

Supplement: Supplementary file 2 — (GIF 103 kb). [file 12035_2018_995_Fig7_ESM.gif]

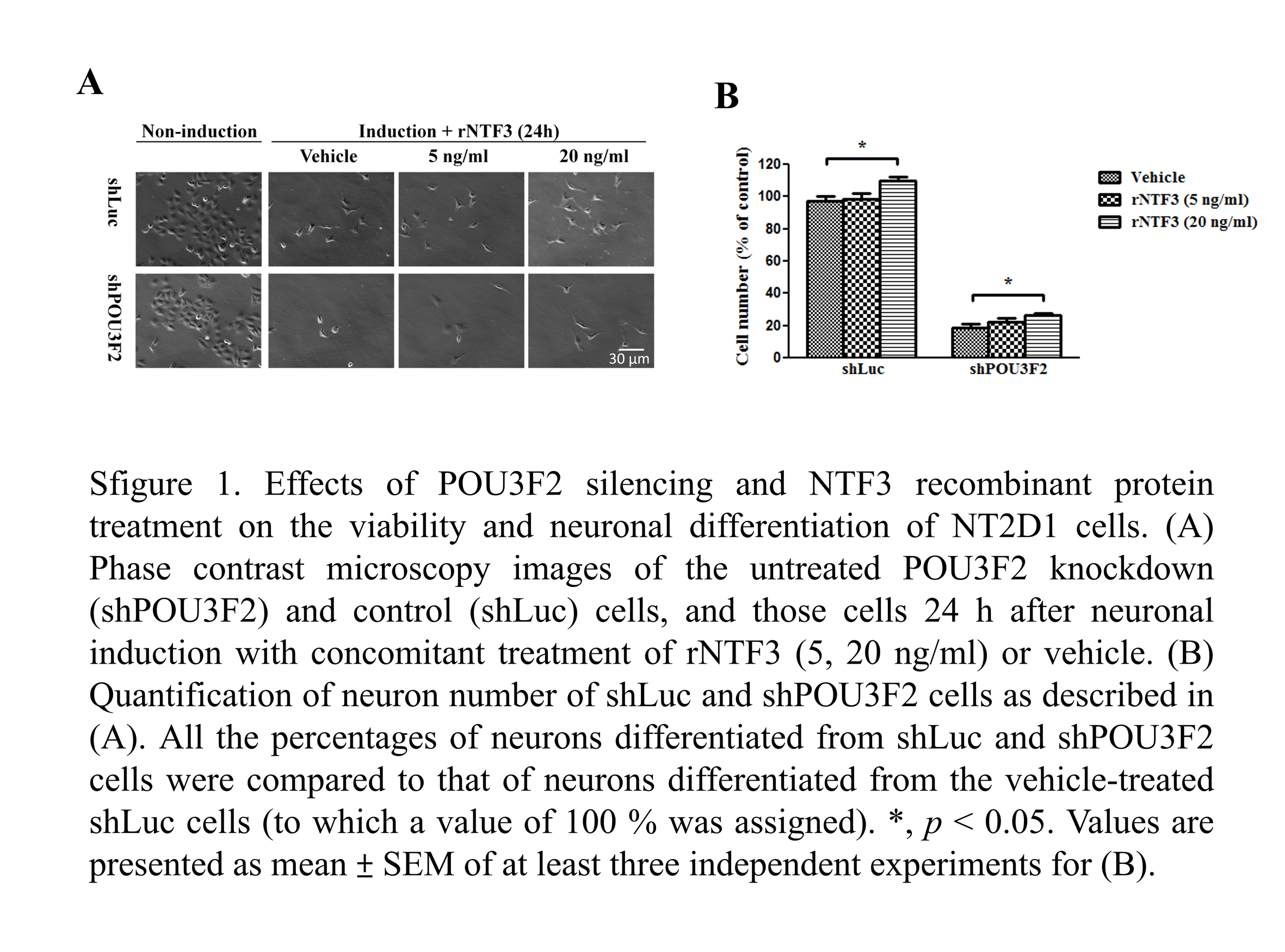

Supplement: Supplementary file 3 — High Resolution Image (TIFF 16170 kb). [file 12035_2018_995_MOESM2_ESM.tif]

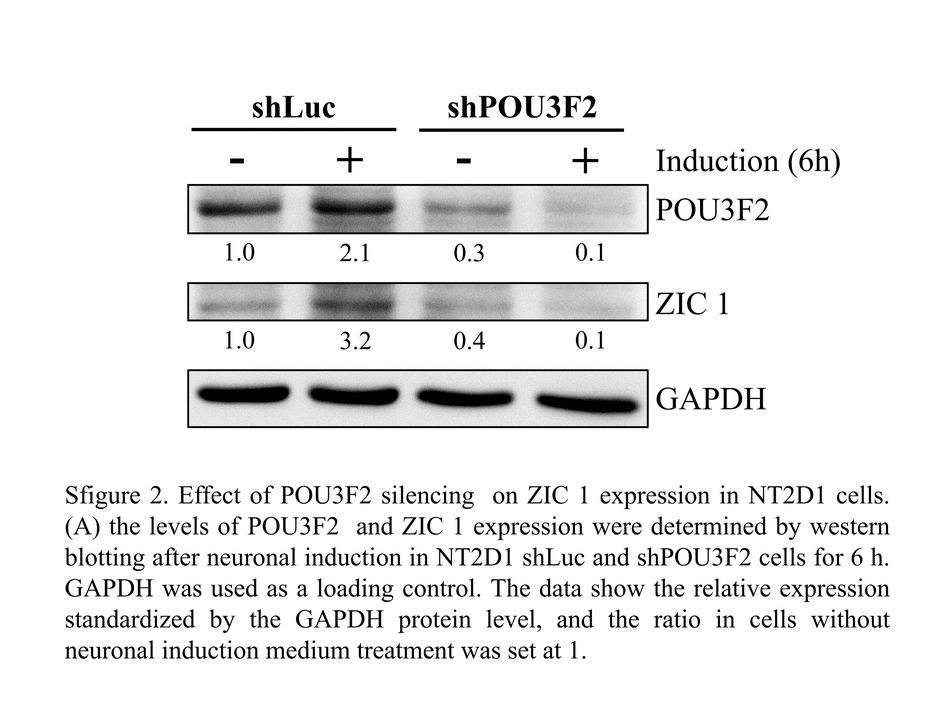

Supplement: Supplementary file 4 — (GIF 67 kb). [file 12035_2018_995_Fig8_ESM.gif]

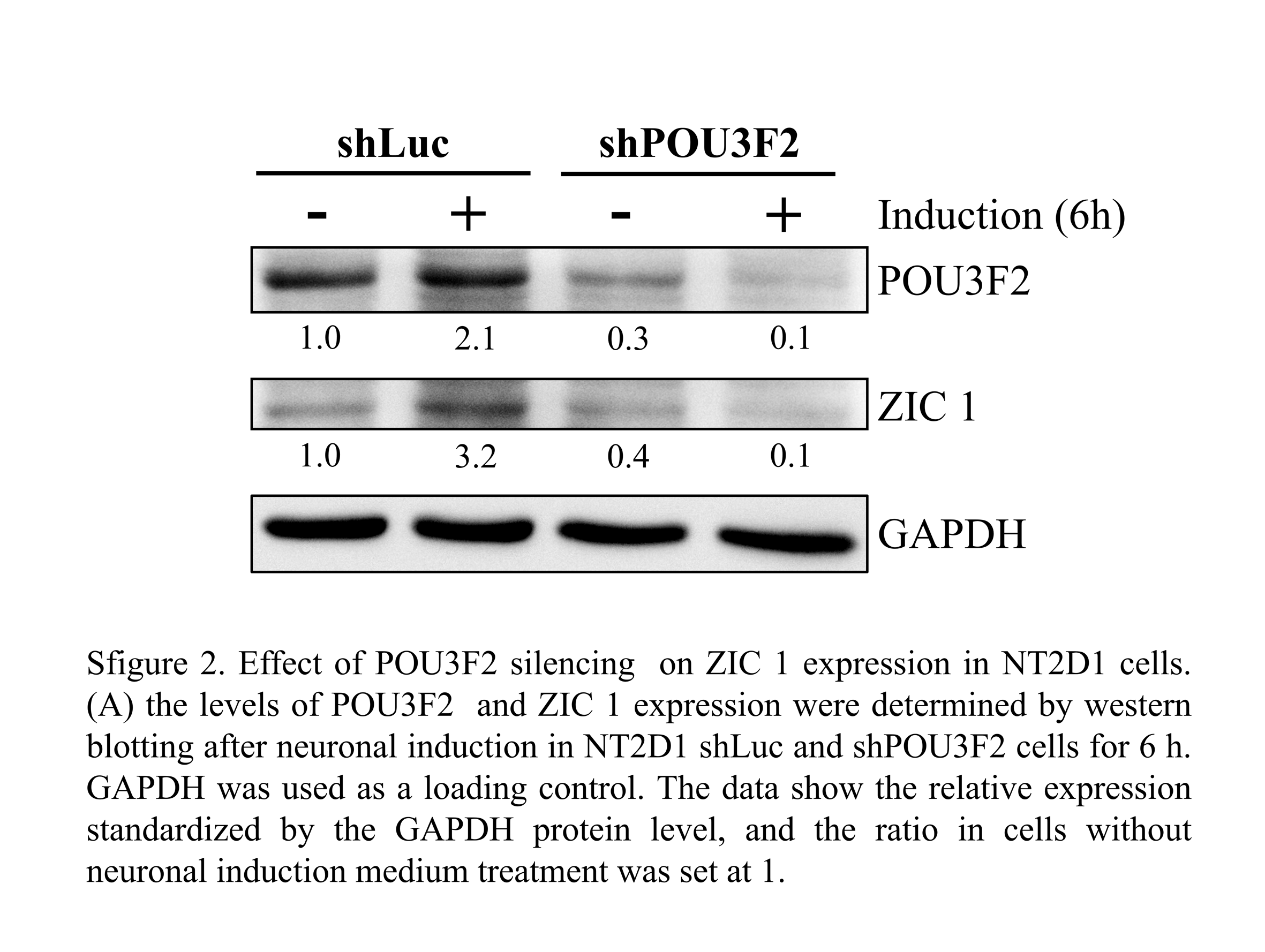

Supplement: Supplementary file 5 — High Resolution Image (TIFF 16427 kb). [file 12035_2018_995_MOESM3_ESM.tif]
